# Optimization of fermentation parameters to improve the biosynthesis of selenium nanoparticles by *Bacillus licheniformis* F1 and its comprehensive application

**DOI:** 10.1186/s12866-024-03410-5

**Published:** 2024-07-20

**Authors:** Zhangqian Wang, Nana Li, Xin Zhou, Shiya Wei, Ying Zhu, Mengjun Li, Jue Gong, Yi He, Xingxing Dong, Chao Gao, Shuiyuan Cheng

**Affiliations:** 1 National R&D Center for Se-rich Agricultural Products Processing, Wuhan, 430028 China; 2 School of Modern Industry for Selenium Science and Engineering, Wuhan Polytechnic University, Wuhan, 430028 China; 3 Medical Department of Gaoming Hospital of TCM, Foshan, 528500 China; 4 Hubei National Se-rich Technology Development Co., Ltd., Enshi, 445000 China

**Keywords:** *Bacillus licheniformis*, Sodium selenite, SeNPs, RSM

## Abstract

**Background:**

Selenium nanoparticles (SeNPs) are increasingly gaining attention due to its characteristics of low toxicity, high activity, and stability. Additionally, *Bacillus licheniformis*, as a probiotic, has achieved remarkable research outcomes in diverse fields such as medicine, feed processing, and pesticides, attracting widespread attention. Consequently, evaluating the activity of probiotics and SeNPs is paramount. The utilization of probiotics to synthesize SeNPs, achieving large-scale industrialization, is a current hotspot in the field of SeNPs synthesis and is currently the most promising synthetic method. To minimize production costs and maximize yield of SeNPs, this study selected agricultural by-products that are nutrient-rich, cost-effective, and readily available as culture medium components. This approach not only fulfills industrial production requirements but also mitigates the impact on downstream processes.

**Results:**

The experimental findings revealed that SeNPs synthesized by *B. licheniformis* F1 exhibited a spherical morphology with diameters ranging from 110 to 170 nm and demonstrating high stability. Both the secondary metabolites of *B. licheniformis* F1 and the synthesized SeNPs possessed significant free radical scavenging ability. To provide a more robust foundation for acquiring large quantities of SeNPs via fermentation with *B. licheniformis* F1, key factors were identified through single-factor experiments and response surface methodology (RSM) include a 2% seed liquid inoculum, a temperature of 37 ℃, and agitation at 180 rpm. Additionally, critical factors during the optimization process were corn powder (11.18 g/L), soybean meal (10.34 g/L), and NaCl (10.68 g/L). Upon validating the optimized conditions and culture medium, *B. licheniformis* F1 can synthesize nearly 100.00% SeNPs from 5 mmol/L sodium selenite. Subsequently, pilot-scale verification in a 5 L fermentor using the optimized medium resulted in a shortened fermentation time, significantly reducing production costs.

**Conclusion:**

In this study, the efficient production of SeNPs by the probiotic *B. licheniformis* F1 was successfully achieved, leading to a significant reduction in fermentation costs. The exploration of the practical applications of this strain holds significant potential and provides valuable guidance for facilitating the industrial-scale implementation of microbial synthesis of SeNPs.

## Introduction

Selenium is an essential element necessary for the normal life activities of the body [1]. It plays an important role in maintaining redox balance, immune regulation, antagonizing heavy metals, inhibiting cancer, and protecting DNA and chromosomes from oxidative damage [2–4]. However, the deficiency of selenium in the human body can also cause diseases, such as Keshan disease, osteosynthesis, and diabetes, etc. [5–7]. The human body must obtain selenium from the diet to meet its needs, but the amount of selenium intake is affected by environmental factors such as soil, water, and food [8]. The bioavailability of different forms of selenium is different. The bioavailability of inorganic selenium is low and the safety range is narrow. It can easily produce toxicity [9], followed by organic selenium, and elemental selenium is the least toxic. SeNPs, as a form of selenium, have high bioavailability and naturally low toxicity compared with inorganic selenium and organic selenium [10]. SeNPs have been demonstrated to possess antiviral, antioxidant, antibacterial, and anticancer properties [11–16]. The research findings reveal that many bacteria, fungi, actinomycetes, and archaea all possess the ability to reduce sodium selenite to synthesize SeNPs. Among these, SeNPs with the smallest particle size, and the particle size is inversely correlated with their biological activity [17]. Therefore, bacteria are typically used as the main agents for the biological synthesis of SeNPs. Bacteria-synthesized SeNPs also exhibit antioxidant and antitumor activities [18, 19]. Previous study conducted antioxidant activity and cytotoxicity tests on MCF-7 cell lines using SeNPs with particle sizes ranging from 80 to 220 nm, prepared from *Bacillus* sp. MSh-1, and compared the results with those of selenium dioxide [20]. The results showed that at a concentration of 200 µg/mL, the radical scavenging capacities of SeNPs and selenium dioxide were 23.1 ± 3.4% and 13.2 ± 3.1%, respectively. Previous studies reported that SeNPs prepared from *B. licheniformis* JS2 were able to induce necroptosis in PC-3 cancer cells mediated by reactive oxygen species through the activation of tumor necrosis factor. Real-time quantitative PCR analysis showed increased expression of tumor necrosis factor and interferon regulatory factor 1 [21]. *Bacillus* sp. has drawn attention due to its excellent selenium tolerance and ability to synthesize SeNPs. It has been reported that certain bacteria within the *Bacillus* sp., such as *Bacillus subtilis* [22], *Bacillus licheniformis* [23], *Bacillus cereus* [24], and *Bacillus megaterium* [25], as a dominant microbial community in the microbiota of both animals and plants, they are widely present in the intestinal tract of animals. However, it has been reported that some *Bacillus* sp. pose potential safety risks. For instance, *Bacillus cereus* [24], could pose a threat to human health, limiting their application in the field of human health. If a safe and effective method for utilizing *Bacillus* in combination with SeNPs could be found, leveraging both probiotic functions and selenium supplementation, it would have a significant impact on human health and nutrition [26]. To address this research gap and identify a strain capable of safely preparing SeNPs, not only should their selenium-rich capabilities be thoroughly studied, but also a comprehensive assessment of their safety is warranted. A method for safely and effectively utilizing *Bacillus* sp. for SeNPs preparation is anticipated to be discovered.

*B. licheniformis* has beneficial life properties such as providing nutrients to the body, enhancing immunity [27], maintaining intestinal microecological balance [28], etc., and has a wide range of applications in the pharmaceutical and feed industries [29], most importantly, *B. licheniformis* is a new type of microecological agent, which can adsorb heavy metals degrade pesticides [30]. The multiple functions of the *B. licheniformis* not only realize the basic function of probiotics, but also solve many problems faced by humans in a greener and more efficient manner. Usually, *B. licheniformis* does not exhibit hemolytic activity. However, research has revealed that *B. licheniformis* isolated from in the air of the Banani area shows β-hemolysis on blood agar [31]. It is necessary to comprehensively evaluate the safety of *B. licheniformis*.

It is well-recognized that an excess of free radicals in the human body can lead to a range of diseases such as cancer, aging, and heart disease [32]. The secondary metabolites of the strain and SeNPs effectively reduce free radicals by regulating reactive oxygen species (ROS) and glutathione peroxidase (GPx), providing cellular protection and preventing damage. Numerous studies both domestically and internationally have reported the strong antioxidant properties of SeNPs. The antioxidant properties are related to the SeNPs [33]. Research indicates that specific *Lactobacillus* strains possess metabolites with antioxidant characteristics, potentially alleviating oxidative stress in human erythrocytes induced by acrylamide [34]. In this study, the antioxidant ability of the secondary metabolites of *B. licheniformis* was determined by measuring the clearance rates of DPPH, ABTS, and hydroxyl radicals. Simultaneously, the antioxidant activity of SeNPs reduced by the strain and its secondary metabolites was tested, aiming to broaden the comprehensive application prospects of *B. licheniformis* and explore the life, health, and beneficial properties of SeNPs.

The majority of research on microbial fermentation of SeNPs is concentrated on the optimization of cultivation conditions [35], with limited attention given to the optimization of the culture medium. The yeast extract and peptone used in the commonly employed LB basal medium are highly processed and expensive, making them unsuitable for subsequent industrial production. In contrast, the raw materials used in the optimized medium are agricultural by-products, which are inexpensive and readily available. Therefore, there is a need to investigate the optimization of the culture medium to enhance yield and reduce costs. Despite extensive studies on microorganisms capable of high SeNPs production, in-depth research on their practical industrial applications remains relatively limited. Current research on microbial SeNPs production primarily centers on the exploration of high-yield strains and molecular mechanisms, with fewer studies on industrial-scale production. To transform these microorganisms into practical industrial applications, further research, and exploration are required to address challenges in their actual production, demonstrating feasibility and economic benefits. Specifically, it is crucial to address the issue of SeNP yield while simultaneously reducing costs. Most studies remain at the shake flask level, and pilot-scale studies, as a crucial platform between laboratory achievements and workshop production, play a key role in the development and optimization of fermentation processes. The poor mixing, unbalanced nutrition, and issues such as bacterial settling in small-scale shake flask fermentations affect experimental results. In contrast, fermentor-scale fermentations can overcome these problems associated with shake flask fermentations. Therefore, research on pilot-scale amplification is essential for the development and optimization of fermentation processes. Many studies focus on selecting strains with high reduction rates, but there is limited research on how to completely convert sodium selenite during the cultivation process [36–38]. Research suggests a close correlation between the biomass of the strain reproducing per unit time and the reduction rate of SeNPs. Therefore, maximizing the reduction rate of SeNPs can be achieved by using more affordable agricultural by-products as raw materials and providing the most suitable culture conditions and medium for the growth and reproduction of the strain through single-factor testing and RSM, enhancing the feasibility of industrial SeNPs production.

This study delved into the practical application prospects of *B. licheniformis* F1 and the SeNPs synthesized by it. Through single-factor experiments and RSM optimization, low-cost large-scale production of SeNPs was achieved, providing strong support for their comprehensive utilization.

## Materials and methods

### Supplies and chemicals

Sodium selenite was purchased from Sigma (Darmstadt, Germany), and tryptone, yeast extract, and NaCl, was purchased from SCR in Shanghai city, China. 2,2-Diphenyl-1-picrylhydrazyl (DPPH, ≥ 95.0%), 2,2′-Azino-bis-3-ethylbenzthiazoline-6-sulfonate (ABTS, ≥ 95.0%), Salicylic acid, and FeSO_4_ were purchased from Sinopharm Chemical Reagent Co., Ltd., Shanghai, China.

### Isolation and purification of strains capable of synthesizing SeNPs

Strains were isolated from the serial dilutions of the soil samples which collected from Zhongshan, Guangdong (N22°49′, E112°31′), China, and dispersed on the Luria-Bertani (LB) plate, culture at 37 ℃ for 24 h, select a single colony and subculture. Prepare 100 mmol/L sodium selenite (Sigma-Aldrich Chemical Co., Milan, Italy), LB medium, and inoculate the purified single colony into the plate, make sure that the colony turns red and is in good condition after 24 h, thi*s* is a strain with a high reduction of sodium selenite. After isolation, a high-concentration-sodium selenite tolerant strain named F1 is obtained.

### Identification of th*e* strain

#### 16S rDNA sequencing and phylogenetic tree

16S rDNA genes were obtained through PCR using the isolated genomic DNA as templates and a pair of universal primers, 27 F (5’-AGAGTTTGATCCTGGCTCAG-3’) and 1492R (5’-GGTTACCTTGTTACGACTT-3’) [39]. The amplified PCR products were then purified by agarose gel electrophoresis, followed by their direct sequencing at the Sheng gong Bioengineering (Shanghai) Co., Ltd. Subsequently, the 16S rDNA sequences were identified in the NCBI database (https://blast.ncbi.nlm.nih.gov/Blast.cgi) by BLAST search and phylogenetic tree analysis via the MEGA 7.0 software.

#### Morphological identification of strain F1

Select a single colony activated on LB medium and transfer it to LB liquid medium, shook the medium at 180 rpm for 12 h at constant temperature of 37 ℃, with an OD_600_ of about 0.8, and prepare the seed solution. Dilute the solution by 10^6^ times and evenly spread it on a LB agar plate and incubate it at constant temperature of 37 ℃. Examine the morphology of the bacterial body in the colony with the diluted bacterial solution under an electron microscope. Similarly, the seed solution was diluted 10^3^ times and spread evenly on an LB agar plate containing 50 mmol/L sodium selenite, incubated at 37 ℃, and observed the morphology of the colony in the sodium selenite plate. The results were compared with “*Bergey’s Manual of Systematic Bacteriology*” Vol. 2 for sugar fermentation, indole, oxidase and citrate utilization experiments [40].

### Growth of strain F1 in different concentrations of sodium selenite

The tests were conducted using the modified method by previous studies [41]. Prepare LB liquid medium with different concentrations of sodium selenite at 0, 50, and 150 mmol/L. The seed solution (The single colony selected from LB medium was inserted into LB liquid medium and culturing in shaker at 37 ℃ for 12 h. After secondary transfer fermentation, the seed liquid with OD_600_ value of 1.0 ± 0.05 was obtained.) was inoculated into a triangular shake flask containing a 100 mL of medium at 1% of the inoculum. The cultured was shake*n* at a constant temperature of 37 ℃ and 180 rpm. Prepare 5 sets of replicates for each sample. Take samples every 2 h, measuring the absorbance value of the bacterial liquid at a wavelength of 600 nm, and draw a growth curve.

### Analysis of the maximum concentration of sodium selenite tolerenc*e* by strain F1 in liquid medium

Due to differences in the ability to generate SeNPs under liquid medium cultivation, we tested the tolerance of strain F1 in LB liquid medium containing different concentrations of sodium selenite. Inoculate selenium tolerant strain F1 seed solution and incubate 1% of the inoculum into LB liquid medium containing 50, 100, 150, 200, 250, 300 mmol/L sodium selenite, at 37 ℃ and 180 rpm in a constant temperature shaker for 48 h, observe the bacterial growth and production status of SeNPs.

### Characterization of SeNPs

The seed solution was inserted into 100 mL LB liquid medium with 1% inoculum amount, 180 rpm, at a constant temperature of 37 ℃ shaking culture to the logarithmic growth phase, 100 mmol/L sodium selenite was added, and after 6 h of culture, centrifuged at 10,000 rpm for 30 min [42]. The precipitate was collected, washed three times with physiological saline, and then add electron microscope fixing solution for Scanning electron microscope (SEM) scanning [43].

Inoculate the activated strain seed liquid according to 1% of the inoculum into LB liquid medium. The medium should contain a certain concentration of sodium selenite and be cultured at a constant temperature of 37 ℃ with shaking at 180 rpm until the fermentation broth turns red. The fermentation broth containing SeNPs is collected in a centrifuge tube, place it in a centrifuge at 10,000 rpm for 30 min. The mixture of bacteria and SeNPs was precipitated. Wash the precipitate with sterile water 2 to 3 times. Place the precipitate in a mortar, add liquid nitrogen and grind immediately; suspend the grounded bacterial powder in sterile water, and sonicate the cells on ice for 6 to 10 min; pass the SeNPs suspension through 20, 10, 5, 3, 1.2, 0.8 μm filter membrane respectively. Transfer the SeNPs suspension to a separatory funnel, add 1/5 volume of, mix well, stand still, and collect the lower layer to be the product; centrifuge at 10,000 rpm for 30 min, collect the precipitate, and wash it with sterile water for 2 to 3 times. Take the precipitate to obtain SeNPs [44].

The optical characteristics of SeNPs were detected using UV-Vis spectroscopy analysis in the wavelength range of 200–800 nm. Elemental analysis of SeNPs was conducted using Energy dispersive spectroscopy analysis (EDS) [43]. The functional groups present in the sample were determined using Fourier-transform infrared (FTIR) analysis by observing the absorption peaks and wavelengths in the infrared spectrum. Purified SeNPs were diluted with potassium bromide at a ratio of 1:100, and the samples were prepared into pellets. All measurements were performed in the range of 400–4000 cm^− 1^ [45]. SeNPs powder was spread on glass slides and analyzed using X-ray diffraction (XRD) [46], with a working voltage of 40 kV, a current of 40 mA, a scanning speed of 10°/min, and a scanning range of 10–80° to determine the crystalline phase in the sample.

### Stability analysis of SeNPs

A batch of SeNPs was prepared. To study the time stability of SeNPs, they were stored at 20 ℃ for 3 d. The pH stability of SeNPs over time was analyzed by adjusting the pH of SeNPs to 1, 3, 7, 9, and 11 using 1 mol/L HCl and 1 mol/L NaOH (test after processing for 4 h on the 0th day). The temperature stability of SeNPs was studied by placing them at 4, 20, 37, and 60 ℃ (test after processing for 4 h on the 0th day). During the experiment, the particle size and zeta potential of SeNPs were measured using dynamic light scattering (DLS) [47].

### Hemolytic activity analysis

*B. licheniformis* F1 were subcultured in LB medium and streaked on LB agar plates containing 5% sheep blood, then incubated for 24 h at 37 ℃, the formation of the transparent layer around the colony was observed to assess the hemolytic activity of the strain [48].

### Antibiotic susceptibility analysis

The sensitivity of strains to antibiotics was detected by Kirby-Bauer (K-B) disk method specified in Clinical and Laboratory Standards Institute (CLSI) standard [49]. 100 µL of *B. licheniformis* F1 seed solution was added to the LB agar plates with a thickness of 4 mm, and the plate was coated evenly. After drying, the plates were pasted with ciprofloxacin, chloramphenicol, ofloxacin, gentamicin, kanamycin, tetracycline, streptomycin and erythromycin, and 3 antibiotic sensitive papers were applied evenly on each plate. One was a blank aseptic paper, then incubated for 24 h at 37 ℃. The diameter of the bacteriostatic zone (mm) was measured. The drug sensitivity standard referred to the CLSI 2014 version.

### Antioxidant activity determination of SeNPs and secondary metabolites

#### Extraction of secondary metabolites and SeNPs of strain F1

The extraction method has been slightly modified based on the previously reported method [22]. The fermentation broth containing SeNPs is collected in a centrifuge tube, placed in a centrifuge at 10,000 rpm for 30 min. Collect the supernatant, extract it using a 1:1 ratio of ethyl acetate, and collect the ethyl acetate layer. After rotary evaporation and weighing to obtain secondary metabolites, absolute ethanol is added to prepare a solution of secondary metabolites.

#### Antioxidant activity determination

##### DPPH assay

The tests were conducted using the modified method by previous studies [50]. Pipette 600 µL of 0.25 mmol/L DPPH (Phygene, Fujian, China) solution (alcohol-free configuration). Then, respectively add 70 µL of 10 mg/mL SeNPs solution or secondary metabolite alcohol solution, distilled water to make up to 670 µL. React in the dark for 30 min, and measure the absorbance at 517 nm. The DPPH radical inhibition rate was calculated as:

$${\rm{DPPH}}\;{\rm{radical}}\;{\rm{inhibition}}\;{\rm{rate}}\% = {{{{\rm{A}}_{\rm{b}}} - ({{\rm{A}}_{\rm{a}}} - {{\rm{A}}_{\rm{b}}})} \over {{{\rm{A}}_{\rm{b}}}}} \times 100$$

Where $\:{\text{A}}_{\text{a}}\:$is the absorbance value after mixing the sample and DPPH solution, $\:{\text{A}}_{\text{c}}$ is the absorbance value after mixing the sample and DPPH solution, $\:{\text{A}}_{\text{b}}$ is the absorbance value after mixing DPPH and water.

##### ABTS assay

The tests were conducted using the modified method by previous studies [51]. Draw up 600 µL of ABTS working solution (0.2 mL ABTS + 0.2 mL K_2_S_2_O_8_ (at room temperature in the dark for 12 h), dilute with absolute ethanol, and control the absorbance at 734 nm to be around 0.7), and add 10 mg/mL SeNPs solution or secondary metabolite alcohol solution 70 µL, distilled water to make the volume to 670 µL, react in a 37 ℃ incubator for 30 min, and measure the absorbance at 734 nm. The ABTS radical inhibition rate was calculated as:

(${\rm{ABTS}}\;{\rm{radical}}\;{\rm{inhibition}}\;{\rm{rate}}\% = {{({{\rm{A}}_0} - {{\rm{A}}_1})} \over {{{\rm{A}}_0}}} \times 100$ is the absorbance value measured by replacing the sample with water; $\:{\text{A}}_{1}$ is the absorbance value after the sample is mixed with the ABTS solution.

##### Hydroxyl assay

The tests were conducted using the modified method by previous studies [52]. In the test tube, add 200 µL of 9 mmol/L FeSO_4_ solution and 200 µL of 9 mmol/L salicylic acid-ethanol solution, respectively. Then, add 10 mg/mL SeNPs or 70 µL of active secondary metabolite solution, distilled water to make up to 470 µL, and 30% 200 µL of H_2_O_2_ solution, let it stand for 30 min, and measure the absorbance at 510 nm wavelength. Calculate the hydroxyl radical inhibition rate using the same formula as described for the ABTS radical inhibition rate above.

Where $\:{A}_{0}$ is the absorbance value measured by replacing the sample with water; $\:{A}_{1}$ is the absorbance value after the sample is mixed with the solution.

### Optimization of fermentation conditions in shaking flask level

Response surface analysis is a statistical mathematical method used to reflect the optimal corresponding conditions obtained when the interaction between various factors in a multi factor system reaches its maximum response value [53]. The biomass of the strain is positively correlated with the reduction rate of SeNPs. By optimizing the fermentation conditions of the strains, using LB medium to optimize the single factor of inoculum size, shaking speed and temperature, culture for 18 h, measure the number of colonies, and explore the optimal fermentation conditions for the number of viable bacteria. Subsequently, from the perspective of the medium, in order to better realize the industrialized fermentation production, the raw materials of the medium are planned to be low-cost, easy-to-obtain, and nutrient-rich agricultural and sideline products. Therefore, a Plackett-Burman design (PBD) with *N* = 7 was employed, involving six factors: corn meal, soybean meal, KCl, peanut cake, NaCl, and bran wheat. These factors were studied, with one being designated as a dummy variable for error estimation. Each factor in the experimental design has two levels. The level of each factor is determined according to the previous single-factor experiment. The high level is 1.6 times the low level. Minitab 17 software is used for experimental design and analysis. Each group of experiments is repeated 3 times, and the results are averaged. According to the results of variance analysis of the PBD experiment revealed three key factors for the subsequent experiment. The positive and negative effects of factors were determined according to the t-value, and the subsequent steepest climbing experimental design was carried out. The follow-up response surface experiment was designed with the optimal values from the climbing experiment as the center values. The Box-Behnken design (BBD) was designed with minitab 17 software and the data were processed to obtain the optimal medium ratio.

### Verification of viable bacteria in shaking flask level

The above MRS optimization results can be analyzed and calculated by minitab 17 software, the best ratio can be obtained and the maximum number of living bacteria can be predicted, 250 mL shake flask was used to verify under the optimized fermentation conditions.

In the 250 mL shake flask, the amount of liquid 50 mL was filled, and the seed liquid was inserted into the basic and optimized LB culture medium commonly used in the laboratory at 2% inoculum. After being cultured in a constant temperature shaker at 37 ℃ and 180 rpm for 24 h, the number of living bacteria was calculated using the previously described method. Then, the fermentation broth was diluted, 30 µL was coated on the LB medium, and the plate colonies were observed after being cultured for 18 h at 37 ℃.

### Verification of SeNPs production in shaking flask level

Selenium standard curve drawing: the concentration gradient of sodium selenite was set to 2, 4, 6, 8, 10 mmol/L, each concentration was 15 mL, and then 25 mmol/L hydroxylamine hydrochloride (NH_2_OH·HCl) was added to each bottle, so that SeO_3_^2−^ was completely converted into Se^0^, and the reaction was fully concentrated by rotary evaporation, and the temperature was set to 40 ℃. After steaming, 3 mL of 1 mol/L Na_2_S solution was added to each reaction bottle, and the absorbance of the solution at 500 nm was determined after 1 h of reaction [54]. Finally, the standard curve was drawn with absorbance value as Y axis and selenium quality as X axis.

In the 250 mL shake flask, the amount of 50 mL was filled, and the seed liquid was inoculated in the 50 mL LB liquid medium containing 5 mmol/L sodium selenite and the optimized medium according to 2% inoculation amount, and cultured in a constant temperature shaker of 180 rpm at 37 ℃. The mixture was centrifuged for 10 min, and the precipitate was collected at a speed of 12,000 rpm every 24 h. Wash the precipitate with 1 mol/L NaCl, centrifuge 10 min with 12,000 rpm, add 3 mL Na_2_S to the precipitate, mix it and set it for 1 h, then 12,000 rpm centrifuge 10 min, collect the supernatant, measure the absorbance value at 500 nm, and finally calculate the selenium content according to the standard curve formula.

$$\eqalign{& {\rm{Sodium}}\,{\rm{selenite}}\,{\rm{reduction rate }}\% \cr & \,\,\,\,\,\,\,\,\,\,\,\,\, = {{SeNPs{\rm{\ }}content{\rm{\ }}in{\rm{\ }}the{\rm{\ }}product} \over {initial{\rm{\ }}selenium{\rm{\ }}treatment{\rm{\ }}content}} \times 100 \cr}$$

### Verification and scale-up of SeNPs production in 5 L fermentor

The optimized culture conditions were as follows: seed liquid inoculation 2%, culture temperature 37 ℃, stirring speed 180 rpm, medium formula: corn meal 11.18 g/L, soybean meal 10.34 g/L, NaCl 10.68 g/L. In a 5 L fermentor, 3 L of culture medium was added and sterilized at 121 ℃ for 20 min. Subsequently, a concentration of 5 mmol/L was achieved by introducing sodium selenite. Due to the high protein content in the culture medium, 0.3% food-grade polyether defoamer was added for the scaled-up validation of SeNPs production. Under the optimized conditions and culture medium, *B. licheniformis* F1 compared the reduction rate of sodium selenite in 250 mL shake flask and fermentor, the method is the same as above.

### Statistical analysis

Each experiment was repeated three times, and data were represented as mean values ± SD. SPSS software (v.18.0) were adopted for statistical analysis (*p* < 0.05). Figures in the present study were plotted by GraphPad prism (v.8.0.1) software.

## Results and discussion

### Strain identification

**Fig. 1:**
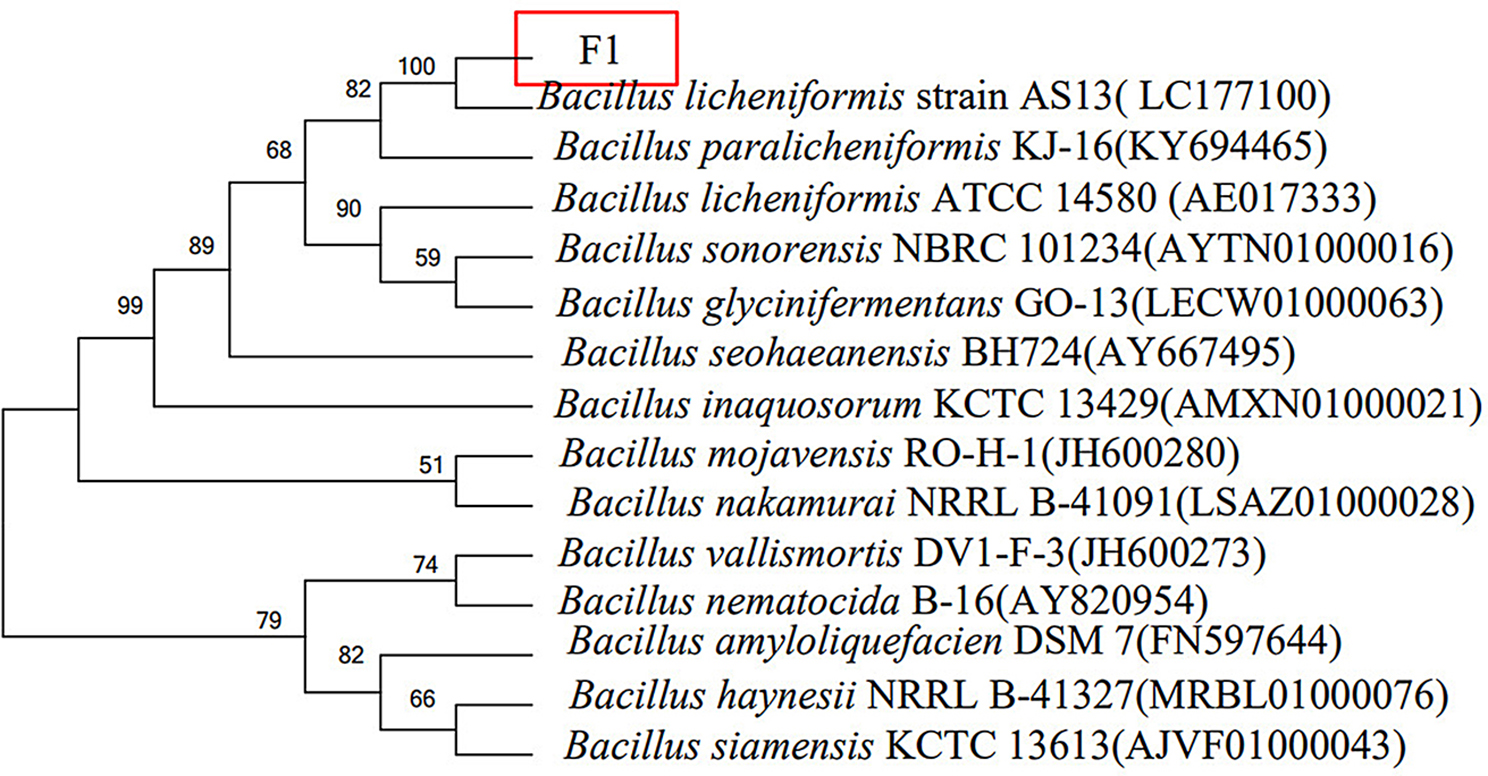
Phylogenetic dendrograms of the studied strains based on 16S rDNA sequencing

**Fig. 2:**
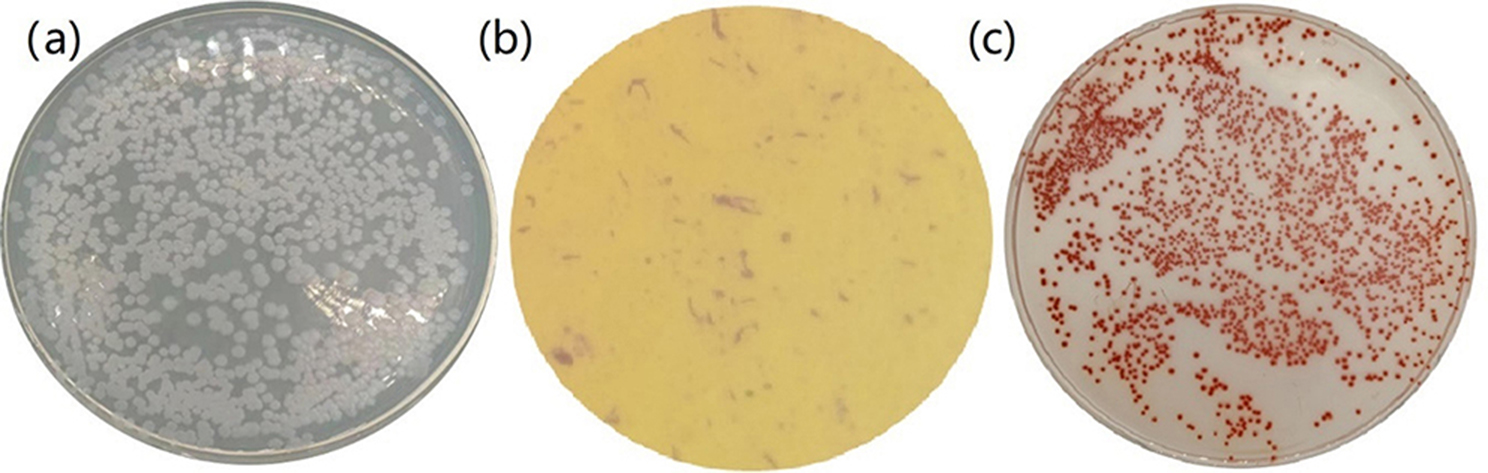
Strain F1 colony morphology on LB agar plate **(a)**, strain F1 morphology in electron microscope **(b)**, strain F1 colony in the form of sodium selenite plate **(c)**

The 16S rDNA sequence of the strain was obtained, and BLAST software was used for gene sequence analysis. It was found that the similarity between strain F1 and *B. licheniformis* strain AS13 was the highest 96.48%, based on their 16S rDNA. The phylogenetic tree constructed by the sequence is shown in Fig. 1. According to the test results, it is preliminarily confirmed that the strain F1 isolated in this strain is *B. licheniformis*. The 16S rDNA sequence of strain F1 was submitted to GenBank with accession number of PP345872. Moreover, the colony of strain F1 is dark round having a rough surface, the mucus is shiny and showed hill-like protrusions (Fig. 2a). Microscopic examination results showed that the bacterial cells were short rod-shaped, single or two arranged side by side into long strips, spores were oval and enlarged in the middle or to one end (Fig. 2b). The colonies on the sodium selenite plate are smooth, round, and red, with a sticky luster, and the size of the colonies is significantly smaller than that on the LB plate (Fig. 2c). Physiological and biochemical results are shown in Table 1. Comparison of “*Bergey’s Manual of Systematic Bacteriology*” accords with the physiological and biochemical characteristics of *B. licheniformis*. Morphological and microscopic observation of strain F1 together with the phylogenetic analysis revealed that strain F1 was identified *Bacillus licheniformis* and named as *B. licheniformis* F1.

**Table 1:** Physiological and biochemical identification results

Experiment	Results	*B. licheniformis*
Glucose	+	+
Sucrose	+	+
Lactose	-	-
Mannose	+	+
Arabic candy	+	+
Indole test	-	-
Oxidase	+	+
Citrate utilization	+	+

+ Positive; - Negative

### Determination of strain growth curve

**Fig. 3:**
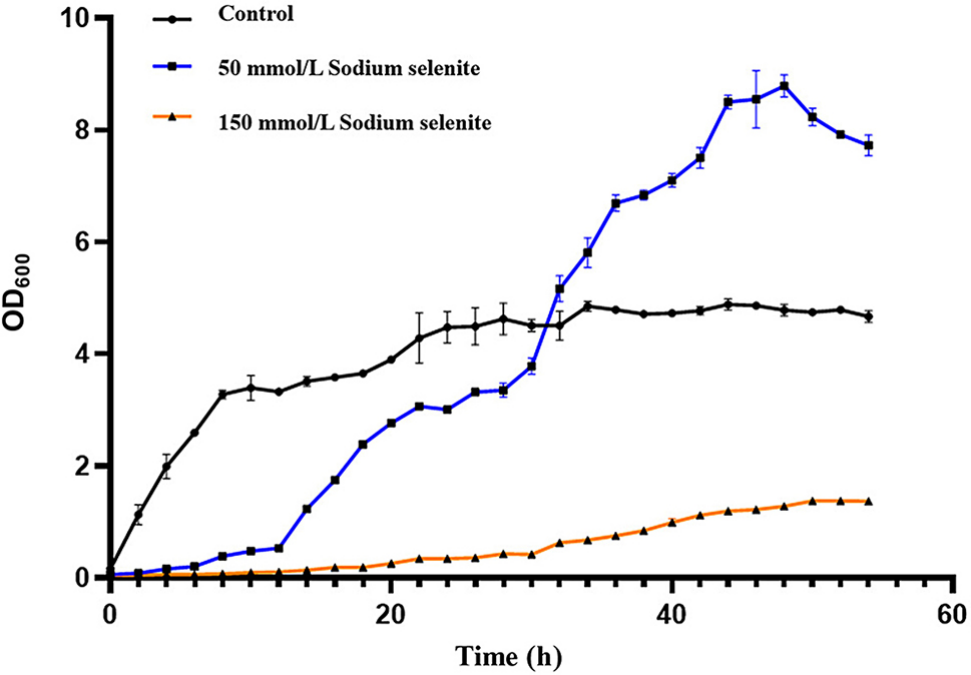
The growth curve of *B. licheniformis* F1 in culture medium with different concentrations of sodium selenite

The growth curve of the strain *B. licheniformis* F1 was shown in Fig. 3. The strain is in the logarithmic growth phase from 3 to 11 h, and enters the stable phase after 23. sodium selenite at a concentration of 50 mmol/L inhibits the growth of the strain. The strain can grow slowly in the high concentration of sodium selenite, enter the logarithmic growth phase after 10 h, and exceed CK after 29 h. This concentration of sodium selenite may promote the growth of the strain. After the 51 h, it quickly enters the decay phase. The strain grows slowly in the sodium selenite concentration of 150 mmol/L. After 43 h, only a certain amount of strains can grow, indicating that high concentrations of sodium selenite will have inhibitory effects on the strains.

### Analysis of the maximum concentration of sodium selenite reduction by strain F1 in liquid medium

**Fig. 4:**
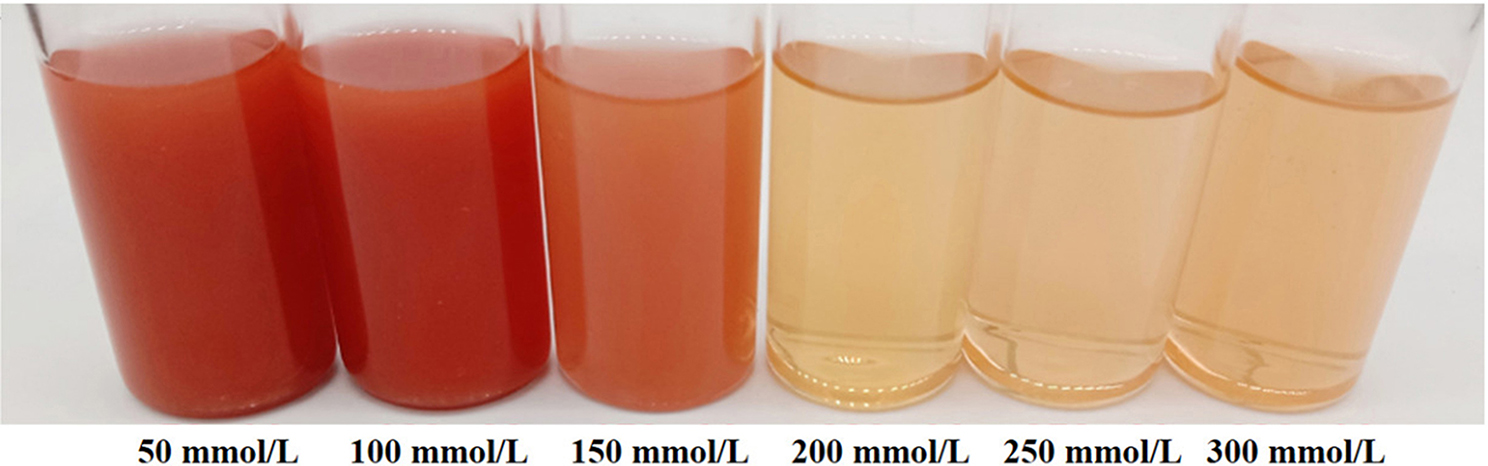
Color changes of strain cultured at different concentrations of sodium selenite for 48 h

The selenium-tolerant strain *B. licheniformis* F1 was cultured in a liquid LB medium containing different sodium selenite concentrations for 48 h. The color changes of the bacteria were shown in Fig. 4 the red color of the culture solution revealed that the strain effectively reduces sodium selenite to red SeNPs up to the maximum of 150 mmol/L sodium selenite concentration. The strain F1’s ability to reduce selenium at ultra-high concentrations makes it useful for treating environmental selenium pollution. It was observed that the color of the bacteria body gradually became lighter with the increase of the sodium selenite concentration in the solution. This indicated that the degree of redness of the bacteria body was negatively correlated with the concentration of sodium selenite in the solution.

### Characterization **of SeNPs**

**Fig. 5:**
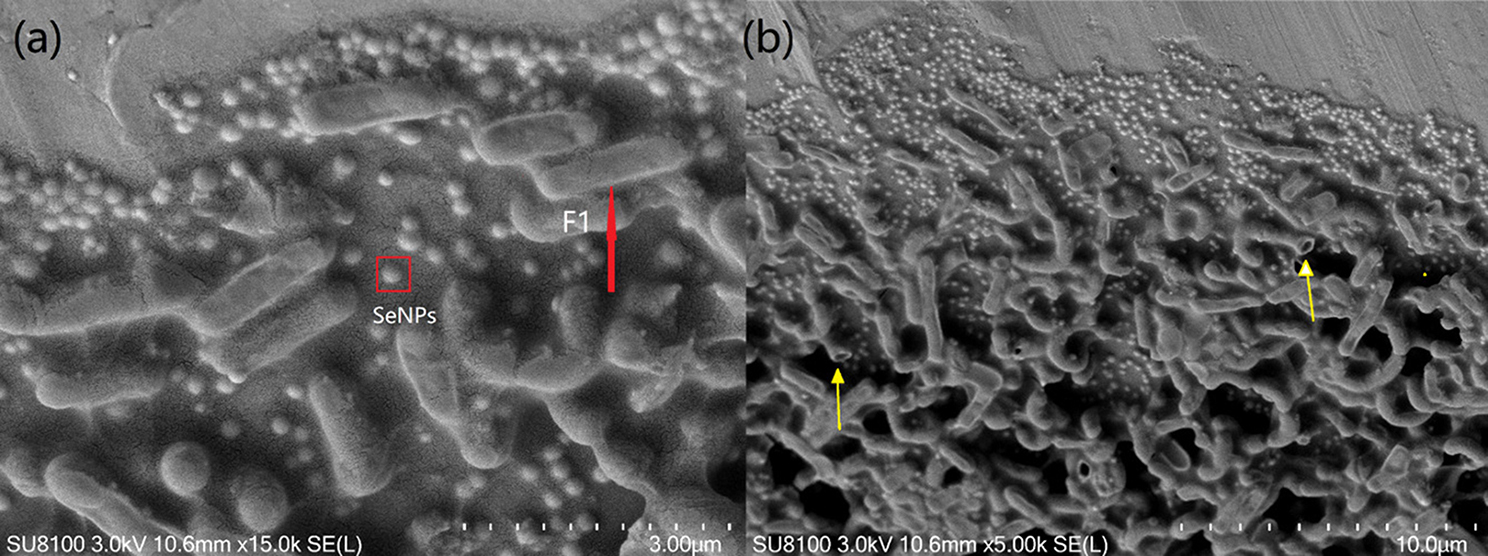
SEM scan of *B. licheniformis* F1 and SeNPs with scale ruler of 3 μm **(a)** and 10 μm **(b)**

To study the effect of sodium selenite exposure on cellular morphology of *B. licheniformis* F1 strain and the production of SeNPs. SEM of different magnifications was performed (Fig. 5), the micrographs obtained showed that *B. licheniformis* F1 was round rod-shaped (red arrow), but under the exposure of sodium selenite, *B. licheniformis* F1 was no longer smooth, and there was a presence of hole structure (yellow arrow), such alterations were presumably caused by the toxic effect of sodium selenite on strain cells. Many particles, with a size of about 110 to 170 nm, were observed outside the strain (red box), indicating the formation of biosynthesized SeNPs.

**Fig. 6:**
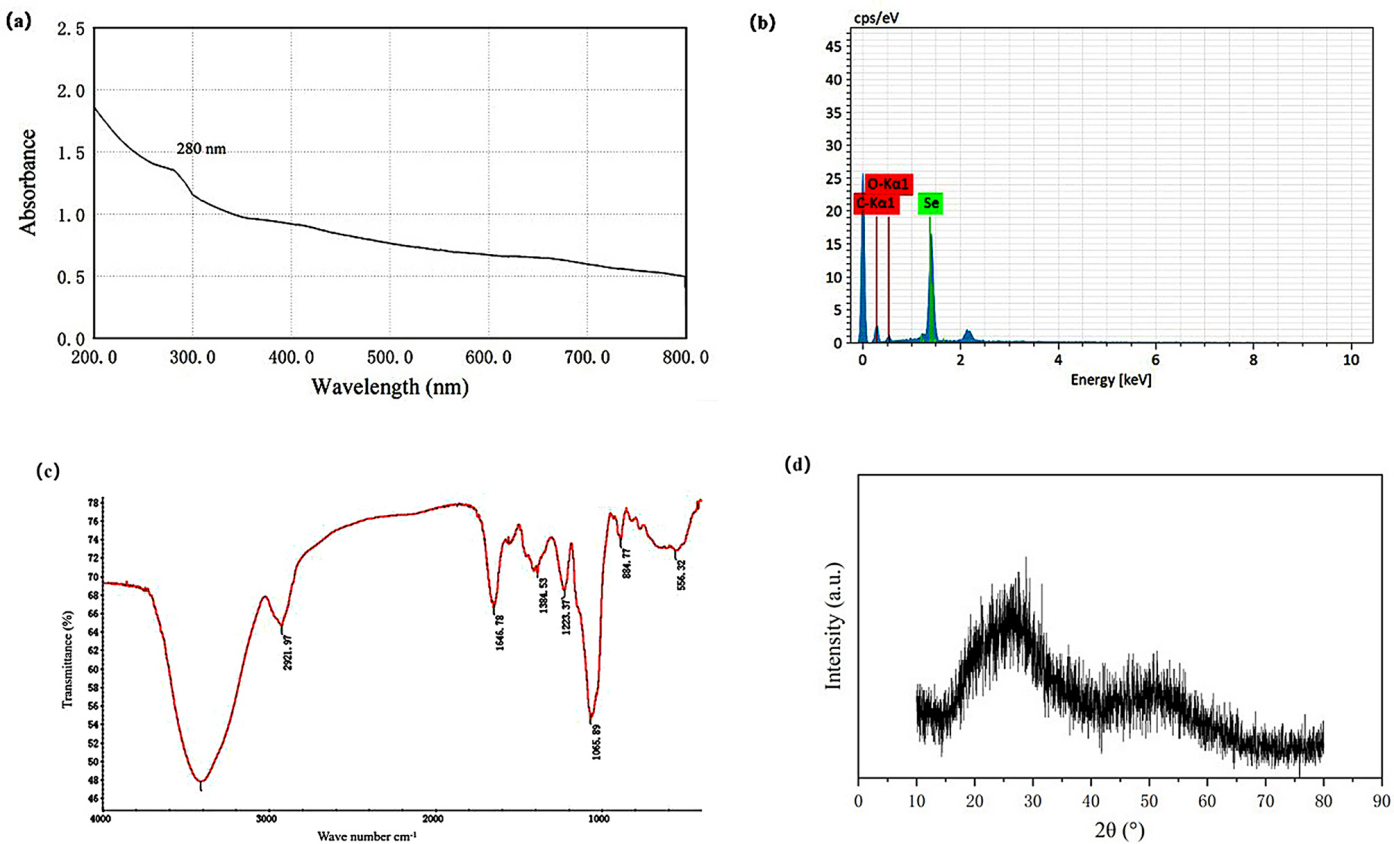
The characterization of SeNPs. UV-Vis spectrum **(a)**, EDS **(b)**, FTIR spectra **(c)**, XRD spectra **(d)**

The UV-Vis spectrum of purified SeNPs showed a peak at 280 nm, typically characteristic of aromatic amino acids, indicating that protein substances were adsorbed on their surface (Fig. 6a). According to the EDS results of SeNPs, the presence of the Se element was confirmed (Fig. 6b), with a selenium weight% of 64.59%. The purified SeNPs reduced by *B. licheniformis* F1 were analyzed by FTIR, revealing characteristic peaks at 3400 cm^− 1^ for O-H stretching vibrations, 2921.97 cm^− 1^ and 884.77 cm^− 1^ for > CH- stretching and C-H bending, 1646.78 cm^− 1^ for tertiary amide, 1384.53 cm^− 1^ and 223.37 cm^− 1^ for COOH/COO- groups, 1065.89 cm^− 1^ for C-N primary amine, and 556.32 cm^− 1^ for C-H stretching vibrations (Fig. 6c). These comprehensive results indicate that the purified SeNPs sample has characteristic peaks of amide, carboxyl groups, etc. It is speculated that the SeNPs contain protein substances. In the XRD spectrum of SeNPs (Fig. 6d), besides the two broad peaks at angles of 20°-30° and 45°-55°, no distinct Bragg reflections were observed, indicating that the synthesized SeNPs are amorphous.

### Stability analysis of SeNPs

Stability is an important factor affecting the functionality and application of SeNPs. In this study, the effects of time, pH, and temperature on the stability of SeNPs were investigated. A small variation in particle size and a zeta potential absolute value greater than 30 mV indicate that the nanoparticles have high stability [55].

**Fig. 7:**
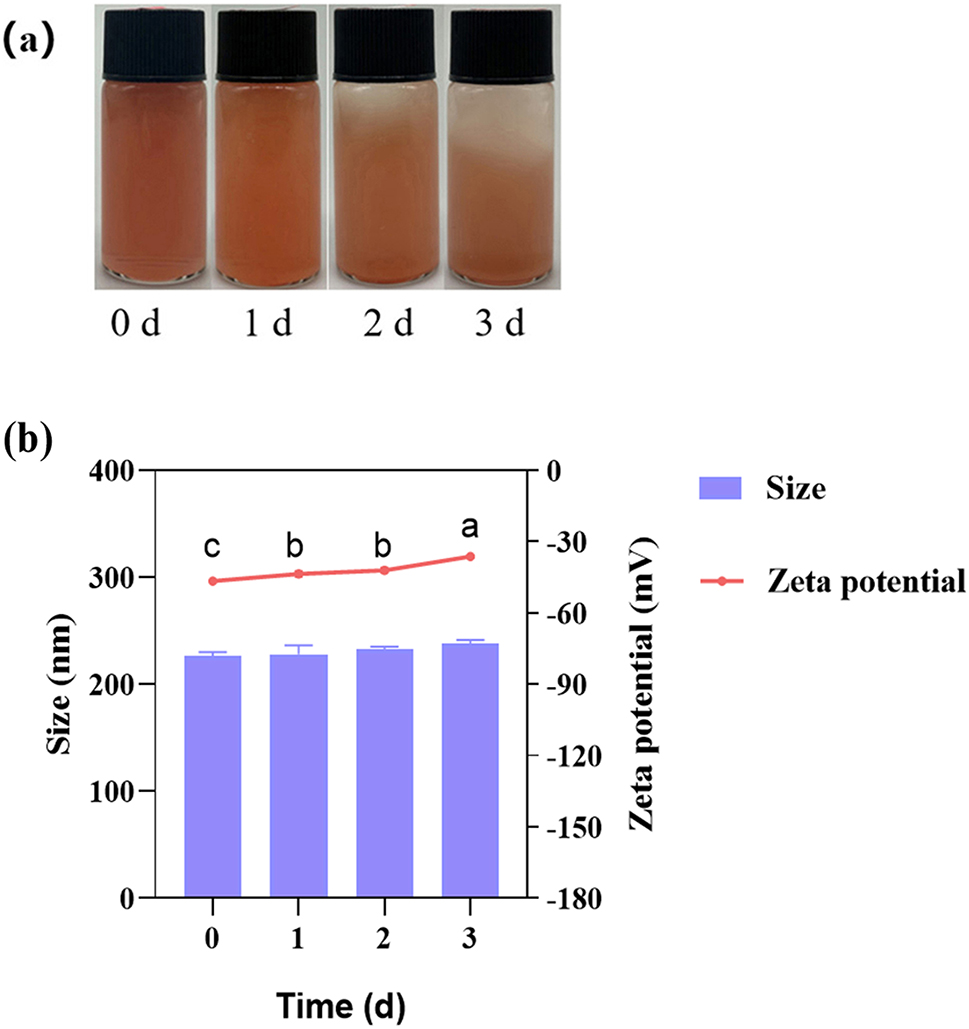
The effect of storage time on the size and stability of SeNPs **(a)**, the particle size and zeta potential of SeNPs at different time **(b)**. The diferent letters (a, b, c, d) represent the signifcant diferences among the media (*p* ＜ 0.05)

There was no significant difference in the particle size of SeNPs after being stored for different days over time, the particle size of SeNPs remained between 221 and 241 nm, indicating their stability (Fig. 7a). When freshly prepared, the size of SeNPs was measured at 226.37 nm, with an electrical potential of -46.59, indicating its high stability. However, the zeta potential showed significant differences, increasing from − 46.59 mV initially to -36.24 mV over time (Fig. 7b).

**Fig. 8:**
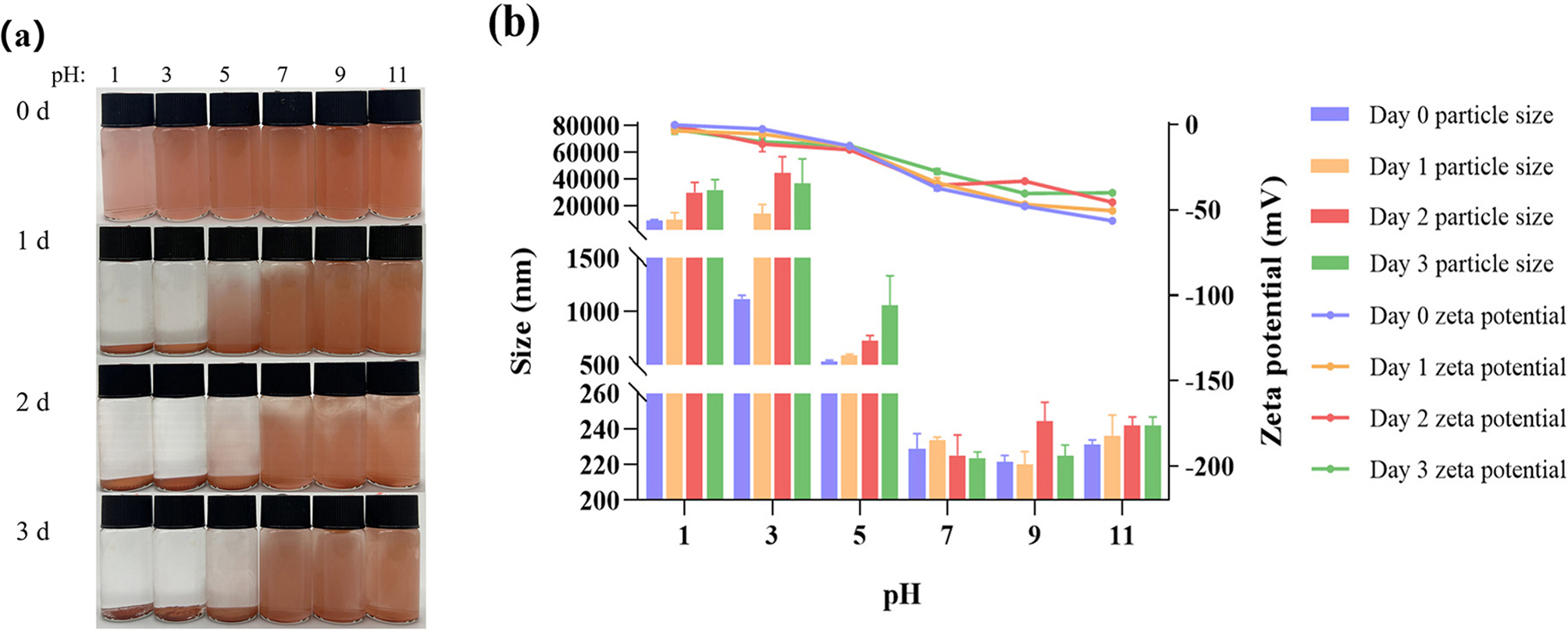
The effect of storage time on SeNPs at different pH **(a)**, changes in particle size and zeta potential of SeNPs at different pH and time **(b)**

The stability of SeNPs treated at different pH levels over time was also analyzed. The freshly prepared SeNPs appeared as a red solution. After 2 d, SeNPs were more stable at pH 7–9 compared to pH 1–5, although some precipitation and instability were observed (Fig. 8a). When the pH is in the range of 7 to 9, the variation in the particle size range of SeNPs is not significant, with zeta potential values below − 30 mV, indicating high stability of SeNPs. This is consistent with the trend observed in the results of SeNPs particle size (Fig. 8b).

**Fig. 9:**
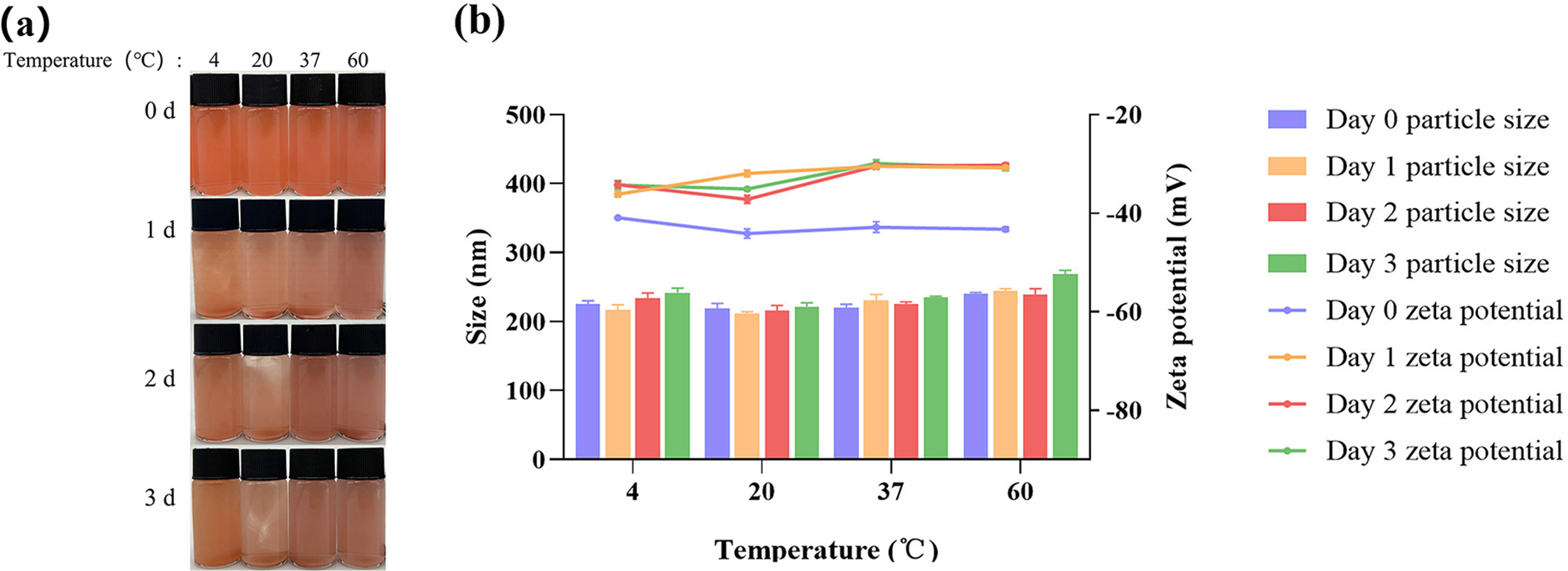
The effect of storage time on SeNPs at different temperatures **(a)**, changes in particle size and zeta potential of SeNPs at different temperatures and time **(b)**

From the macro images, it was evident that SeNPs were most stable at 4 ℃ (Fig. 9a). SeNPs were tested using DLS, and at 4 ℃ and 20 ℃, the zeta potential values were below − 30 mV, indicating high stability of the SeNPs. However, when the temperature was increased to 60 ℃, the SeNPs aggregated significantly, and the particle size increased from 240 nm to 270 nm (Fig. 9b).

### Hemolytic activity analysis

**Fig. 10:**
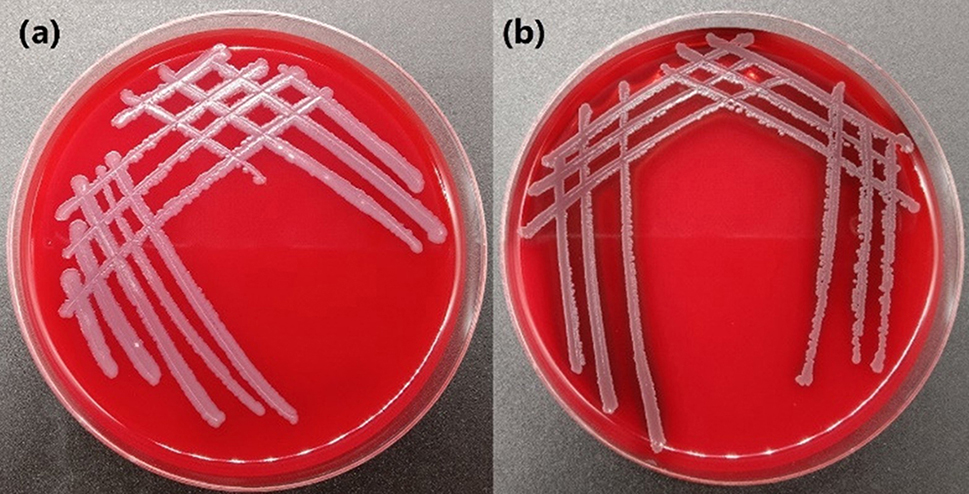
Growth morphology of *B. licheniformis* F1 **(a)**, certain *Bacillus* sp. with hemolytic properties **(b)** on blood agar plate

On blood agar plates, hemolytic strains exhibit hemolysis, resulting in the appearance of a transparent layer, as illustrated in the figure. *B. licheniformis* F1 was cultured on blood agar plates, and no transparent layer or hemolytic activity was observed (Fig. 10a). The bacterium shown in the figure is a strain of *Bacillus* sp. with hemolytic characteristics, which was selected in the laboratory. It displays clear hemolysis on the blood agar plate (Fig. 10b).

### Antibiotic susceptibility analysis

In the laboratory, the disk diffusion method specified in the CLSI standard is usually used to determine the sensitivity of strains to antibiotics according to the diameter of the bacteriostatic zone. In this study, 8 kinds of antibiotics commonly used in the human body were used for drug sensitivity tests. The drug sensitivity standard and the drug sensitivity of *B. licheniformis* F1 were shown in Table 2. The strains were sensitive to ciprofloxacin, chloramphenicol, ofloxacin, gentamicin, and kanamycin, and moderately sensitive to tetracycline and streptomycin erythromycin. The results showed that the strain was sensitive to most common antibiotics and had no obvious drug resistance.

**Table 2:** Results of the antibiotic susceptibility of *B. licheniformis* F1

Antibiotic	Specifications (µg/disk)	Diameter of bacteriostatic zone (mm)			Actual diameter of bacteriostatic zone (mm)	Sensitivity
		*R*	I	S		
Tetracycline	30	≤ 14	15–18	≥ 19	17.61 ± 0.51	I
Ciprofloxacin	5	≤ 15	16–20	≥ 21	30.92 ± 0.12	S
Erythromycin	15	≤ 13	14–22	≥ 23	14.96 ± 0.37	I
Chloramphenicol	30	≤ 12	13–17	≥ 18	21.37 ± 0.19	S
Ofloxacin	5	≤ 13	14–16	≥ 17	29.63 ± 0.84	S
Gentamicin	10	≤ 12	13–14	≥ 15	20.72 ± 0.23	S
Kanamycin	30	≤ 13	14–17	≥ 18	22.06 ± 0.78	S
Streptomycin	10	≤ 11	12–14	≥ 15	14.33 ± 0.16	I

R, resistant; I, intermediate susceptible; S, susceptible

### Antioxidant activity determination of SeNPs and secondary metabolites

**Fig. 11:**
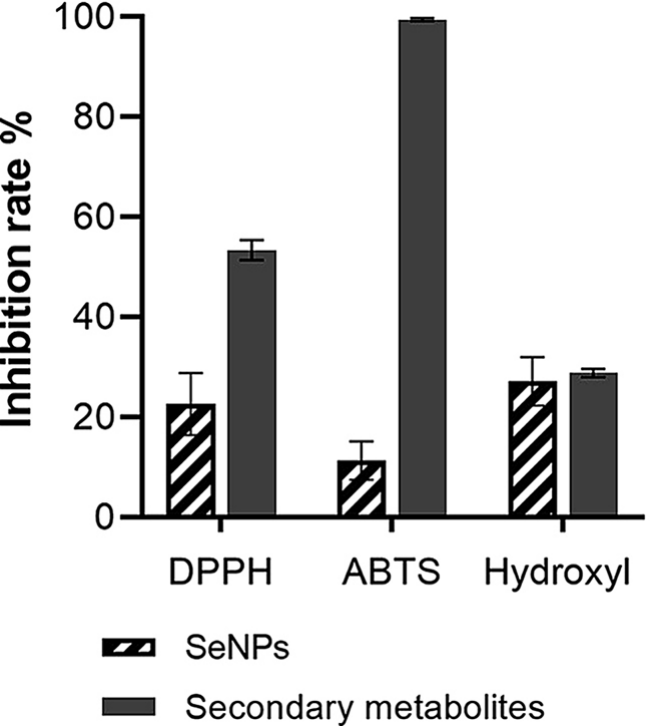
The inhibitory rates of various free radicals by SeNPs and secondary metabolites of *B. licheniformis* F1

The results of the three free radical scavenging rates are shown in Fig. 11. The strain’s ethyl acetate extract and SeNPs both exhibit certain free radical scavenging capabilities. The biological activity of the ethyl acetate extract is relatively high, with the highest inhibition rate observed against ABTS free radicals, reaching 99.69 ± 0.23%. This suggests significant potential for its role as an antioxidant.

### Optimization of fermentation conditions in shaking flask level

**Fig. 12:**
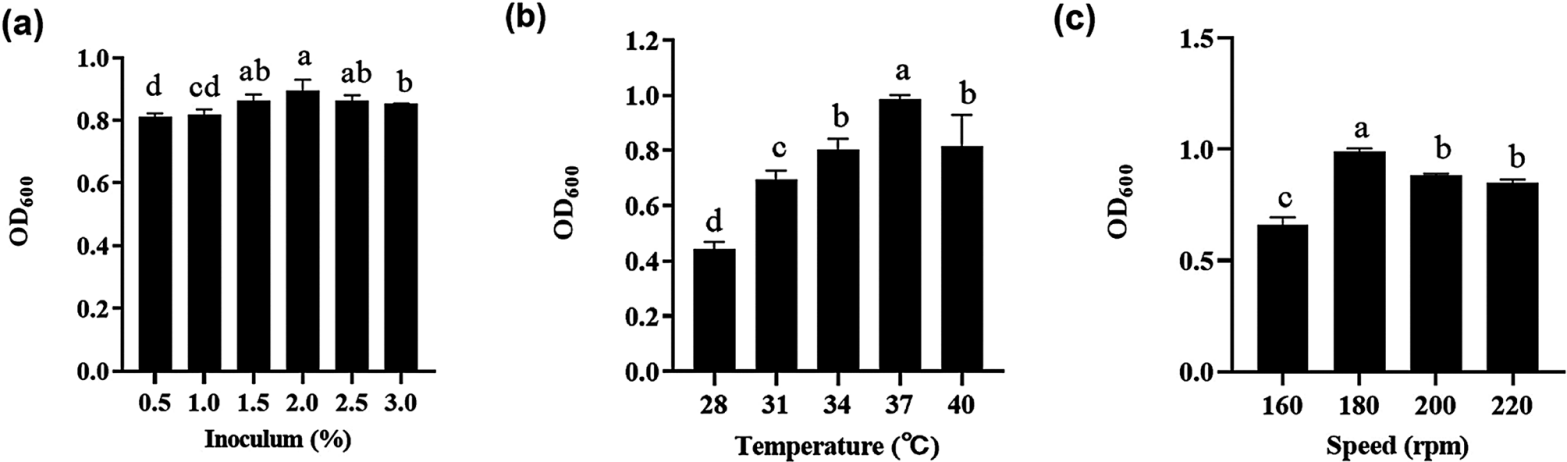
Effect of single factors on the optimum fermentation conditions of *B. licheniformis* F1 inoculum level **(a)**, temperature **(b)**, shaking speed**(c)**

The optimal fermentation conditions for *B. licheniformis* F1 were 2% inoculum, 37 ℃, and 180 rpm (Fig. 12). Therefore, the results were used for subsequent strain medium optimization. According to the variance analysis of the PBD results, it can be seen that when *B. licheniformis* F1 is cultured at high density, the medium factors that significantly impact on the number of viable bacteria are E (NaCl), A (corn meal), and B (soybean meal) in turn (*P* = 0.025, *P* = 0.031, *P* = 0.039). These three factors have been identified as the key factors for the next experiment. The positive and negative effects of factors were determined according to the t-value, and the subsequent steepest climbing experimental design was carried out (Table 3). According to the experimental results of the steepest climbing path, with the increasing concentration of corn meal, soybean meal, and NaCl, the number of viable bacteria of *B. licheniformis* F1 increased first and then decreased. When the content of corn meal is 10 g/L, the content of soybean meal is 10 g/L, and the content of NaCl is 10 g/L, the number of viable bacteria reaches the highest, which is the maximum corresponding value of the three factors. Therefore, the level of each factor in run 4 is the central value of Design follow-up response surface experiments (Table 4). After the optimum concentration range of the three important factors was determined by the climbing experiment, the response surface analysis was carried out. The content of corn meal as 10 g/L, soybean meal content as 10 g/L, and NaCl content as 10 g/L (Table 5). The response surface quadratic regression fitting was performed with Minitab software, and the quadratic polynomial regression equation was obtained:

$$\eqalign{Y &= {\rm{ }}4.967 + 0.459{\rm{X}}1 + 0.026{\rm{X}}2 + 0.898{\rm{X}}3 - 0.055{\rm{X}}1{\rm{X}}2 \cr &+ 0.697{\rm{X}}1{\rm{X}}3 + 0.412{\rm{X}}2{\rm{X}}3 - 1.176{\rm{X}}{1^2} - 0.851{\rm{X}}{2^2} - 0.928{\rm{X}}{3^2} \cr}$$

**Fig. 13:**
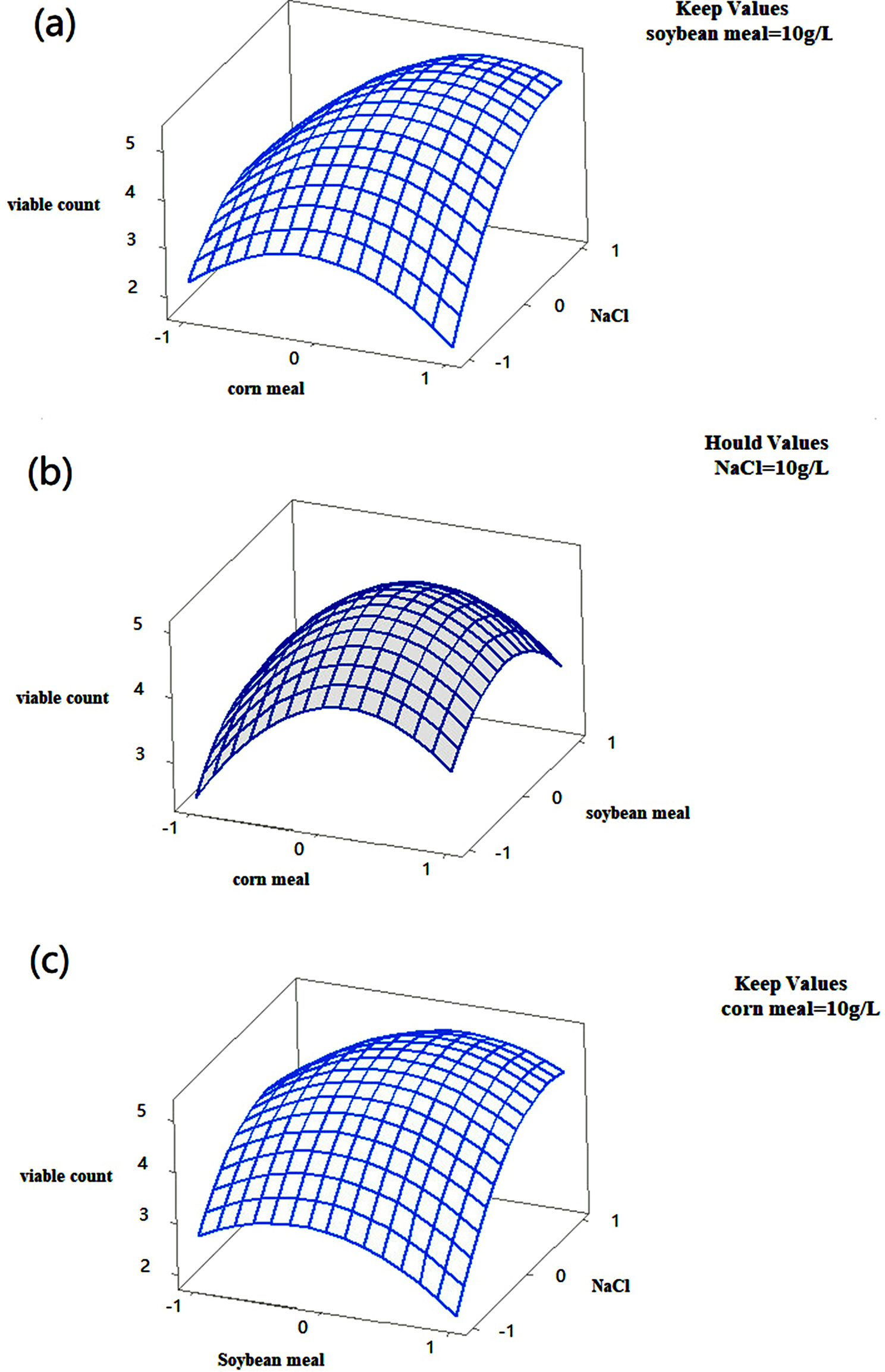
Response surface 3D contour plots representing the interaction between variables affecting viable count corn meal and NaCl **(a)**, soybean meal and corn meal **(b)**, soybean meal and NaCl **(c)**, While the other variable were kept constant and indicated as hold value

The above regression model is analyzed by variance, the regression model *p* = 0.001 (< 0.005), the lack of fit *p* = 0.159 (> 0.005), the model is significant, the lack of fit is not significant, the adjusted correlation coefficient and correlation coefficient of the model are ADJ R^2^ = 94.23% and R^2^ = 97.94%, so the regression equation can be used to predict the experimental results (Table 6). According to the p-value of the three factors, it can be seen that the influences on the number of viable bacteria from large to small are NaCl, corn meal, and soybean meal, which is the same as the previous PBD results. Through the response surface map and contour map, it can be seen that the interaction between corn meal and NaCl, soybean meal and NaCl can find the maximum value (Fig. 13). After the analysis and calculation by the Minitab software, the content of corn meal is 11.18 g/L, and the content of soybean meal is 10.34 g/L, when the NaCl content is 10.68 g/L, there is an optimal value, that is, the maximum number of viable bacteria.

**Table 3:** ANOVA of PBD model (coded units)

Code	Term	*t*-Value	*p*-Value	Order of importance
	Constant	10.22	0.001*	
A	Corn meal	-3.25	0.031*	2
B	Soybean meal	3.03	0.039*	3
C	KCl	1.17	0.306	5
D	Peanut cake	-1.69	0.166	4
E	NaCl	3.48	0.025*	1
F	Bran wheat	0.29	0.784	6

*, *p* < 0.05

**Table 4:** Experimental results of the steepest climbing path

Runs	Corn meal (g/L)	Soybean meal (g/L)	NaCl (g/L)	Viable count (×10^9^ cfu/mL)
1	16	4	4	1.50
2	14	6	6	1.63
3	12	8	8	1.66
4	10	10	10	2.11
5	8	12	12	1.66
6	6	14	14	0.77
7	4	16	16	0.66

**Table 5:** The BBD matrix of three variables and experimental results

Runs	Corn meal (g/L)		Soybean meal (g/L)		NaCl (g/L)		Viable count (×10^9^ cfu/mL)
	Coded	Experimental	Coded	Experimental	Coded	Experimental	Experimental
1	-1	7	-1	8	0	10	2.66
2	-1	7	0	10	-1	9	2.12
3	1	13	1	12	0	10	3.11
4	0	10	-1	8	1	11	3.66
5	0	10	0	10	0	10	5.00
6	-1	7	0	10	1	11	2.33
7	-1	7	1	12	0	10	2.66
8	0	10	-1	8	-1	9	2.50
9	0	10	1	12	1	11	4.70
10	0	10	0	10	0	10	5.10
11	0	10	0	10	0	10	4.80
12	1	13	-1	8	0	10	3.33
13	1	13	0	10	1	11	5.00
14	0	10	1	12	-1	9	1.89
15	1	13	0	10	-1	9	2.00

**Table 6:** ANOVA of the experimental result of the BBD

Source	DF	Adj SS	Adj Ms	f-Value	*p*-Value
Model	9	20.3166	2.25740	26.42	0.001
A	1	1.6836	1.68361	19.70	0.007
B	1	0.0055	0.00551	0.06	0.810
E	1	6.4441	6.44405	75.41	0.000
A^2^	1	5.1049	5.10493	59.74	0.001
B^2^	1	2.6729	2.67293	31.28	0.003
E^2^	1	3.1820	3.18204	37.23	0.002
AB	1	0.0121	0.01210	0.14	0.722
AE	1	1.9460	1.94602	22.77	0.005
BE	1	0.6806	0.68062	7.96	0.037
Lack-of-Fit	3	0.3806	0.12688	5.44	0.159
Pure error	2	0.0467	0.23333		

### Verification of viable bacteria and SeNPs production in shaking flask level

**Fig. 14:**
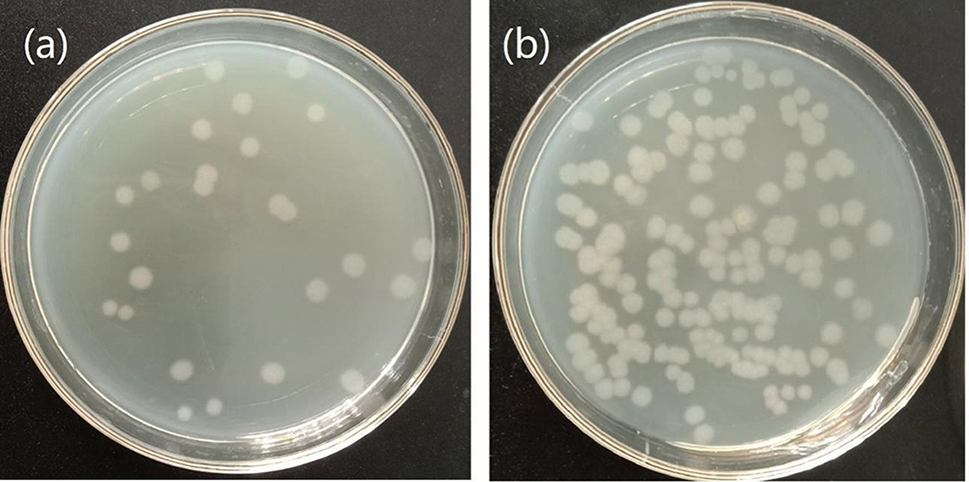
After 24 h fermentation of the two media, the fermentation liquid was diluted and coated with colony diagram LB medium **(a)**, and optimized medium **(b)**

**Fig. 15:**
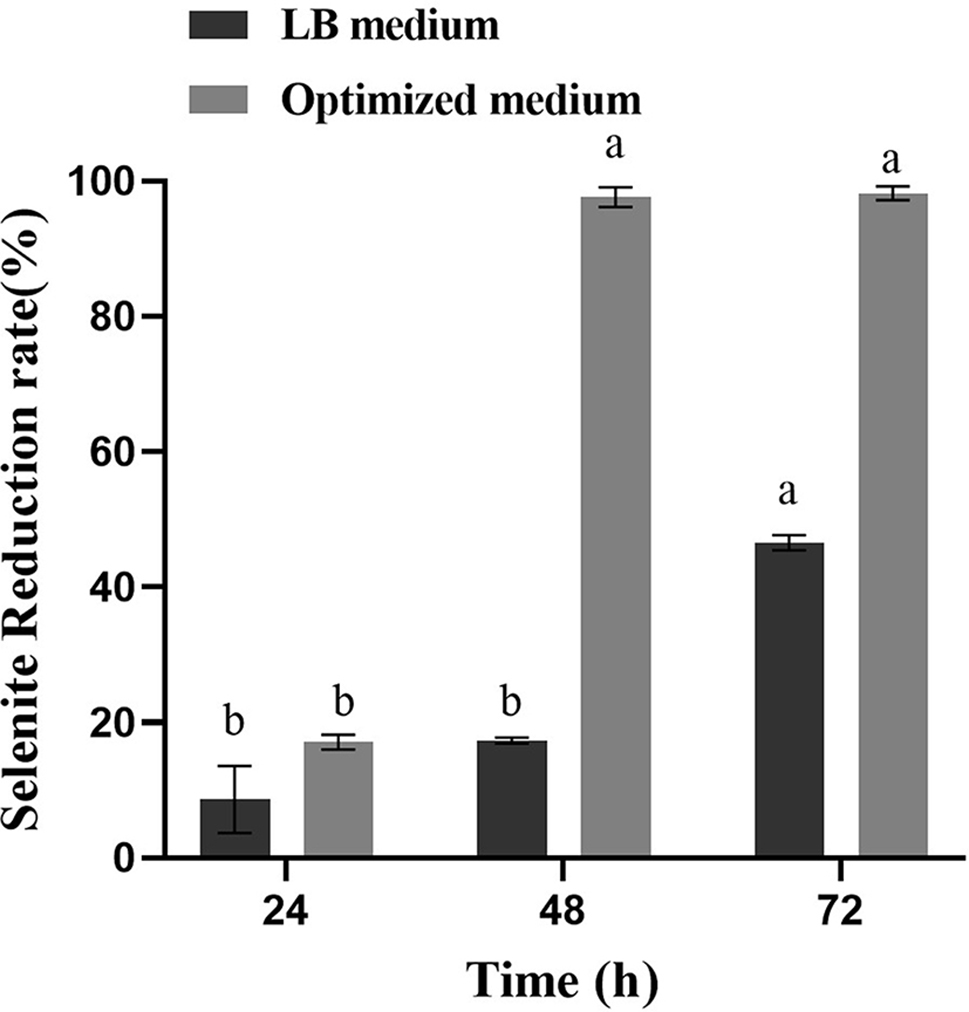
Results of sodium selenite reduction rate in two kinds of culture media

Observing the colony images on agar plates visually confirmed an increase in the cell density of the strain F1 on the optimized culture medium (Fig. 14). Through calculations, it was determined that in the optimized culture medium, the theoretically maximum viable cell count is 5.356 × 10^9^ cfu/mL, while the measured value is 5.532 × 10^9^ cfu/mL. In the optimized medium, the theoretical maximum number of viable bacteria is 5.356 × 10^9^ cfu/mL, and the measured value is 5.532 × 10^9^ cfu/mL. Which has little deviation from the theoretical value, and is higher than that of the LB medium. Based on the experimental results above, the optimized medium successfully increased the number of viable bacteria. Under identical cultivation conditions and initial inoculum levels, *B. licheniformis* F1 in LB alkaline medium exhibited an approximately onefold increase in the reduction rate of 5 mmol/L sodium selenite after validating the optimized conditions and medium (Fig. 15). This substantial enhancement in SeNPs production, coupled with a reduction in medium costs by approximately 93.89%, significantly improves the production capacity. The utilization of low-cost and readily available raw materials makes it more conducive to meeting practical production requirements.

### Verification and scaling up of SeNPs production in fermentor

**Fig. 16:**
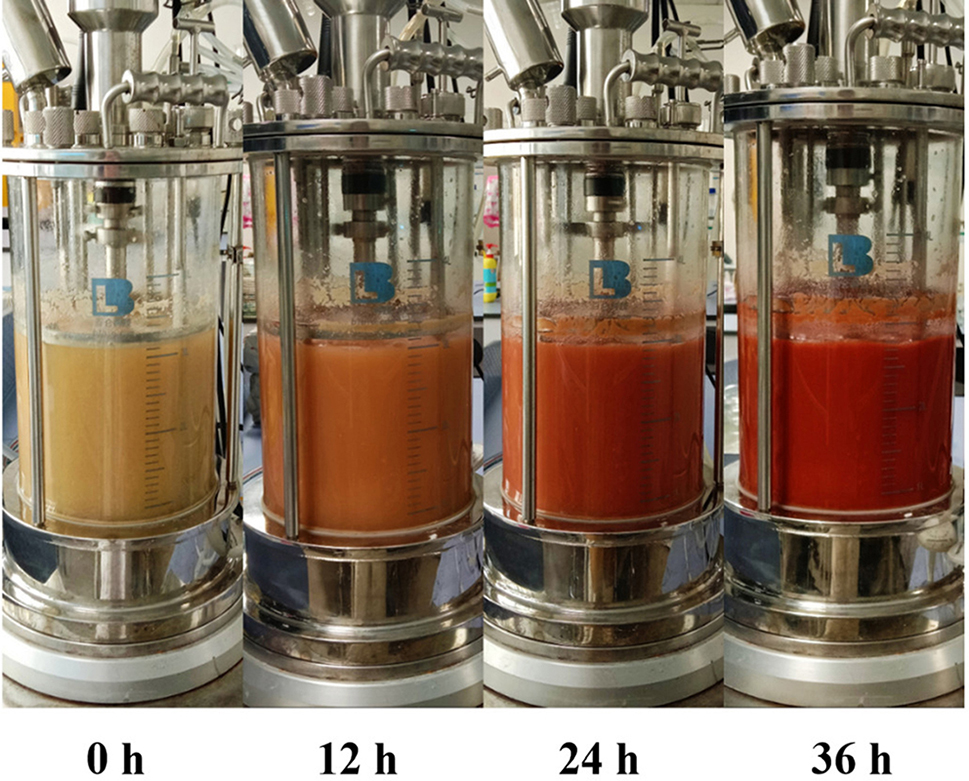
Reduction of sodium selenite to SeNPs by *B. licheniformis* F1 in 5 L fermentor

In the fermentor, the SeNPs of *B. licheniformis* F1 were synthesized in 5 L fermentor using the fermentation conditions and medium obtained from the shaking flask experiment. The process of strain F1 reducing sodium selenite to SeNPs in the fermentor is observable. From the color, it can be seen that the red color in the tank deepens every 12 h. Additionally, the yield of red SeNPs increases gradually (Fig. 16).

**Fig. 17:**
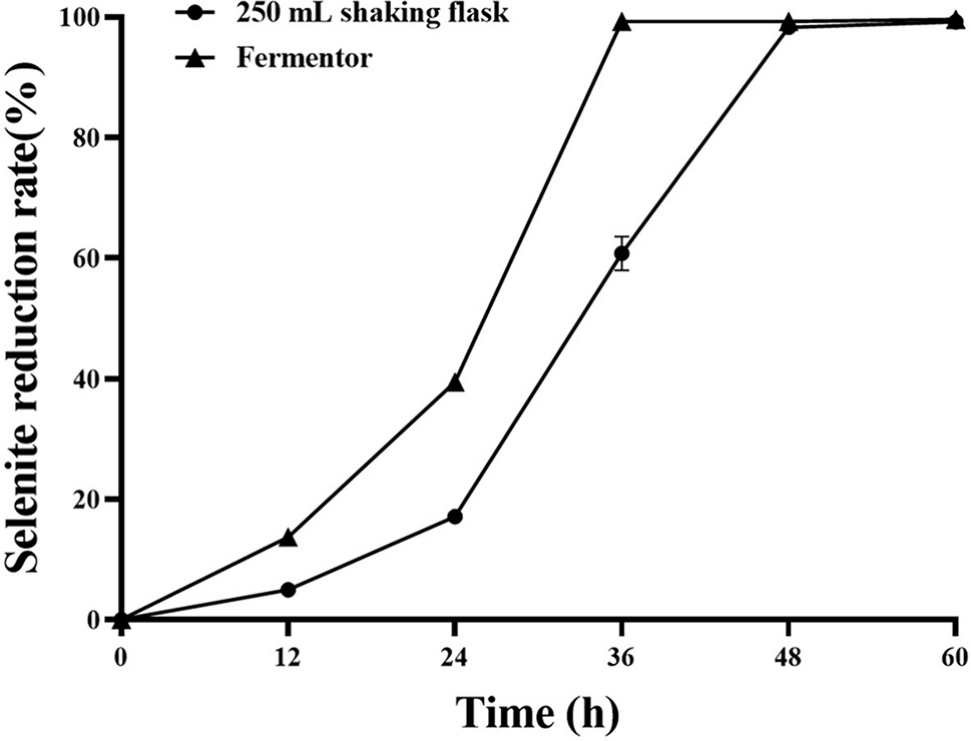
Results of selenium reduction rate of *B. licheniformis* F1 in two kinds of culture media

The samples were taken at intervals, and the sodium selenite reduction rate was compared with that of a 250 mL shaking flask. In the 250 mL shake flask, the reduction rate of sodium selenite of the strain began to increase steadily at 24 h and approached the fermentation endpoint at 48 h. At this time, the reduction rate reached 98.29 ± 0.84%, and almost all reduction was achieved, reaching 99.11 ± 0.07% at 60 h, which was not much different from that at 48 h (Fig. 17).

From the point of view of the reduction rate of sodium selenite, compared with the shaking flask, the fermentation time was shortened and the production efficiency of SeNPs was improved successfully by using the fermentor. The reduction rate of sodium selenite was significantly accelerated, and the fermentation endpoint was reached 12 h ahead of schedule. The reduction rate can reach 99.28 ± 0.57%. The reason may be that compressed air is continuously pumped into the fermentor to maintain positive pressure, and the oxygen content in the tank is high, while *B. licheniformis* F1, as a facultative aerobic bacteria, can accelerate its growth and reproduction with sufficient oxygen. The increase in biomass within the tank accelerates the rate of sodium selenite reduction, and the high oxygen content in the tank may also contribute to the acceleration of the strain’s inherent sodium selenite reduction rate to some extent. In summary, it has been demonstrated that the fermentation conditions and culture medium are applicable for fermentor production, laying the groundwork for the subsequent industrialization of SeNPs.

## Discussion

To prepare SeNPs, the key factor is the screening and evaluation of strains with high tolerance and fast reduction rate of sodium selenite. In this study, the probiotic *B. licheniformis* F1 was isolated from soil samples. Through identification of the maximum selenium tolerance and drawing growth curves of the strain at different concentrations of sodium selenite, it was demonstrated that this strain has strong selenium tolerance. Even in an environment with 150 mmol/L of sodium selenite, it can still reduce SeNPs, indicating a higher selenium tolerance compared to some other reported bacteria such as *Vibrio natriegens* [56] and *Pseudomonas moraviensis* [57], which tolerate 100 and 120 mmol/L sodium selenite, respectively. The SeNPs produced by the strain were characterized, and scanning electron microscope analysis revealed particle sizes ranging from 110 to 170 nm, exhibiting a spherical shape distributed outside the bacterial cells. Kora reported SeNPs synthesized by *Bacillus cereus* AJK3 with sizes ranging from 50 to 150 nm [58], while Hamid isolated a marine *Bacillus* sp. MSh-1 produces SeNPs with sizes ranging from approximately 80 to 220 nm [20]. The surface of the nano-selenium synthesized by *B. licheniformis* F1 is enveloped by proteins, consistent with what is described in the relevant literature. Nano-selenium synthesized by microorganisms is enveloped in a layer of protein, which stabilizes its particle structure uniformly, serving as a natural dispersant, thus possessing good biological activity [59]. Through stability testing, it was found that SeNPs have high stability, maintaining this stability at pH levels of 7–9 and temperatures of 4 ℃ and 20 ℃. These results indicate that *B. licheniformis* F1 can produce small, highly stable SeNPs, providing a versatile platform for biotechnological applications.

Hemolysin secreted by hemolytic strains can make red blood cells dissolve and intracellular components leak out [60]. Hemolysin also acts on a variety of eukaryotic cells, such as cardiomyocytes, endothelial cells, platelet granulocytes, and so on. Toxic effects not only cause local tissue damage, but also cause bacterial infection, vomiting, diarrhea, gastroenteritis and even meningitis, cardiac arrest, and other diseases [61]. Antibiotic resistance is also a crucial component of safety assessments for probiotic strains [62]. If a strain shows resistance to antibiotics, relevant resistance genes may exist on transferable plasmids, increasing the risk of transferring resistance genes to the human genome [63]. This gene transfer, facilitated by gene expression, poses potential dangers. In terms of safety, *B. licheniformis* F1 does not exhibit hemolytic characteristics, and sensitivity testing to common antibiotics shows no significant resistance, thereby reducing the spread of bacterial resistance.

Antioxidants can reduce oxidative stress occurrence [64], and microbial antioxidants have advantages in safety and effectiveness compared to chemically synthesized antioxidants [65]. Therefore, the development of probiotics with antioxidant capabilities and SeNPs is of significant research value. To achieve synergistic probiotic effects between *B. licheniformis* F1, antioxidant capabilities were determined for the strain F1 ethyl acetate extract and its synthesized SeNPs. According to previous studies., it has been demonstrated that chitosan-coated SeNPs can scavenge DPPH, ABTS, and lipid peroxides, and SeNPs can directly scavenge reactive oxygen species [66]. Studies have shown that both ethyl acetate extract of *B. licheniformis* F1 and SeNPs exhibit free radical scavenging activity.

Culture media are composed of different nutrients in specific proportions, serving as the foundation for microbial growth and development [67]. Due to variations in microbial nutrient types and requirements, different microbial growths have diverse practical requirements for culture media. Therefore, optimizing culture media is essential to achieve cultivation goals, control costs, or meet other requirements [68]. To achieve resource utilization and waste reduction, cost control now often utilizes inexpensive and readily available substances such as soybean meal and bran or by-products generated during production processes [69]. Research results indicate that optimal conditions for strain growth and reproduction in the shaking bed stage include a cultivation temperature of 37 ℃, a shaking bed speed of 180 rpm, and a seed liquid inoculation volume of 2%. Through PBD, steepest ascent experiments, and BBD response surface experiments to optimize the culture medium, the optimal formula for strain growth and reproduction was obtained as follows: cornmeal 11.18 g/L, soybean meal 10.34 g/L, and NaCl 10.68 g/L. Using these optimized conditions and culture medium, the reduction rate of 5 mmol/L sodium selenite increased to 99.19 ± 0.05%, and on top of a 93.89% reduction in culture medium cost, the reduction rate almost reached 100%, significantly increasing production efficiency. Fermentation rate determination using a fermentor, compared to shaking flasks, successfully achieved a shorter fermentation time, reaching the end of fermentation by the 36th hour with a reduction rate of 99.28 ± 0.57%. These fermentation conditions and culture medium are suitable for fermentor production, providing fundamental data for large-scale SeNPs fermentation in the later stages.

## Conclusion

In this study, *B. licheniformis* F1 was successfully screened and isolated from the soil, exhibiting its exceptional tolerance to high sodium selenite concentrations and ability to effectively convert it into red-colored SeNPs. Through the research, it was discovered that *B. licheniformis* F1 is considered a safe probiotic. The secondary metabolites of *B. licheniformis* F1, along with the synthesized SeNPs, exhibit a certain level of free radical scavenging activity. This study optimized the fermentation conditions and culture medium for the production of SeNPs by *B. licheniformis* F1 through single-factor experiments and RSM. At the same time, the optimized fermentation conditions were validated in a fermentor. The final results indicate that the reduction rate of sodium selenite doubled, reaching approximately 100%. This method successfully increased the quantity of viable bacteria and significantly enhanced the production rate of SeNPs, enabling low-cost large-scale production of SeNPs. The cost was reduced by approximately 93.89%, providing strong support for its comprehensive utilization. It holds the potential to broaden the application prospects of *B. licheniformis* and provide an economically efficient method for the industrial production of SeNPs.

## Data Availability

Sequence data supporting the findings of this study have been deposited in the NCBI GenBank under the accession code PP345872.

## Abbreviations

SeNPs: Selenium nanoparticles
LB: Luria-Bertani
SEM: Scanning electron microscopy
EDX: Energy dispersive X-ray
FTIR: Fourier-transform infrared
XRD: X-ray diffraction
DLS: Dynamic light scattering
PBD: Plackett-burman design
BBD: Box-Behnken design
RSM: Response surface methodology
